# Assessing constancy of substitution rates in viruses over evolutionary time

**DOI:** 10.1186/1471-2105-11-S6-S3

**Published:** 2010-10-07

**Authors:** Ulrich Melcher

**Affiliations:** 1 Department of Biochemistry & Molecular Biology, Oklahoma State University, Stillwater OK 74078, USA

## Abstract

**Background:**

Phylogenetic analyses reveal probable patterns of divergence of present day organisms from common ancestors.  The points of divergence of lineages can be dated if a corresponding historical or fossil record exists.  For many species, in particular viruses, such records are rare.  Recently, Bayesian phylogenetic analysis using sequences from closely related organisms isolated at different times have been used to calibrate divergences.  Phylogenetic analyses depend on the assumption that the average substitution rates that can be calculated from the data apply throughout the course of evolution.

**Results:**

The present study tests this crucial assumption by charting the kinds of substitutions observed between pairs of sequences with different levels of total substitutions.  Datasets of aligned sequences, both viral and non-viral, were assembled.  For each pair of sequences in an aligned set, the distribution of nucleotide interchanges and the total number of changes were calculated.  Data were binned according to total numbers of changes and plotted.  The accumulation of the six possible interchange types in retroelements as a function of distance followed closely the expected hyperbolic relationship.  For other datasets, however, significant deviations from this relationship were noted. A rapid initial accumulation of transition interchanges was frequent among the datasets and anomalous changes occurred at specific divergence levels.

**Conclusions:**

The accumulation profiles suggested that substantial changes in frequencies of types of substitutions occur over the course of evolution and that such changes should be considered in evaluating and dating viral phylogenies.

## Background

Phylogenetic analyses reveal probable patterns of divergence of present day organisms from common ancestors.  The points of divergence of lineages can be dated if a corresponding historical or fossil record exists.  For many species, in particular viruses, such records are rare.  Substitution frequencies calculated from analysis of strains of single viral species suggest that many viruses are evolving so rapidly that any traces of distant evolutionary events should be obscured [1,2]. On the other hand, evidence has been presented [3-7] that phylogenetic trees of larger taxa often mirror those of their hosts, implying codivergence of virus with host.  This implication is consistent with conclusions from analysis of viral hallmark genes that viruses existed at the time that cellular life evolved [8].  The two views appear to conflict [2,9].  Substitution frequencies required for the codivergence hypothesis need to be about 10^4^ fold less than those observed in many viral species [2,7].  A recent review [10] suggests, by way of reconciliation of the two views, that different evolutionary mechanisms are at work in viral speciation than in the evolution of viral strains of individual species.

Evolutionary theory has recognized that not all residues in a nucleotide or amino acid sequence are subject to the same evolutionary constraints [11].  It also has allowed for the possibility that some kinds of substitutions occur more frequently than others [12] and that overall substitution rates may speed up or slow down along selected lineages [13].  Phylogenetic applications [14,15] allow incorporation of these possibilities for variation into the models to be tested.  One application, DAMBE, allows one to examine the relative rates of accumulation of transitions and transversions [16], but not to distinguish between transitions or among transversions.  However, no phylogenetic model-testing applications allow the individual substitution frequencies to vary relative to one another over the course of evolution.  Constancy is important particularly when phylogenetic trees are constructed and dated using only recent dates as references, such as in BEAST [14].  The present study was undertaken to answer questions about variation of the relative proportions of the different substitution types over evolutionary time.  Do interchanges noted between recently diverged sequence pairs have distributions of interchange types similar to those of distantly diverged pairs?  If they are not the same, do the relative rates of types of interchange change gradually or abruptly over divergence time?  Does coding ability of a strand influence relative rates of types of interchanges?  Are other factors involved?

For this analysis three viral datasets and three non-viral datasets were chosen. Available complete genome sequences of virus species in the *Tobamovirus* genus (family *Virgaviridae*) served as one dataset.  These viruses have single strands of positive sense RNA as their encapsidated genomes.  *Wheat streak mosaic virus* is a single viral species with multiple sequence representatives of moderately wide diversity.  Its genome consists also of a single strand of positive sense RNA.  To contrast with these viruses, the study included isolates of *Tomato yellow leaf curl virus* of which there are several closely related species distinguished by geographic location of original isolation.  The genomes of these viruses are single-stranded circular DNA molecules that have open reading frames on both their genomic and their anti-genomic strands.  A series of eucaryotic retroelements were chosen as coding regions with relaxed evolutionary constraints.  Coding regions for each of the two subunits of ribulose-bisphosphate carboxylase were included since that encoding RbcL is plastid localized and that for RbcS is encoded in the nuclear genome.  Plastid and nucleus are expected to have different mutational profiles.

## Methods

### Datasets

Six datasets were assembled from sequences available in GenBank/EMBL/DDBJ for coding regions for the large subunit of ribulose bisphosphate carboxylase (rbcL) from red algae, the small subunit of the same enzyme (rbcS) from cereals, reverse transcriptase (RT) coding sequences from retroelements of a diversity of sources selected from the EST database, isolates of *Wheat streak mosaic virus* and the related *Oat necrotic mottle virus* (WSMV), members of the *Tobamovirus* genus and isolates of *Tomato yellow leaf curl virus* (TYLCV).  Lists of the accession numbers used are presented in additional file 1.  Sequences were aligned manually using Se-Al [17] with reference to the amino acid sequences encoded.  In the case of tobamoviruses, previously constructed alignments [5] were used as guide.  Alignments are available as fasta format in additional file 2.

### Analysis

Each of the six datasets was analyzed for the numbers of each type of the six interchanges for one strand for each pair of sequences in the dataset and the total numbers of positions evaluated for each pair was recorded. Positions where either member of the pair lacked a residue were ignored.  For datasets with open reading frames on one strand only, that strand was used for analysis.  When ORFs were on both strands, as in TYLCV, the genomic strand was used.  Analysis was accomplished by a short program written in Future Basic (Staz Software, Bay St. Louis, Missouri; source code available as additional file 3). The program tabulated the numbers of each type of interchange in each pair of sequences and the total number of differences in each pair.

Results of the pairwise comparison were imported into Microsoft Excel. The total number of evaluated interchanges, normalized by the number of positions compared were converted to t-distances using the Jukes-Cantor correction for multiple substitutions [18].  For each of the six interchange types, values were  converted to numbers of each type per knt compared. The results were sorted according to t-distances. The numbers of interchanges were then binned according to t-distance to give between 30 and 60 well populated bins. Under-populated bins were removed from consideration.  For each interchange type the mean numbers of interchanges per knt in each bin were plotted as a function of t-distance. For each each t-distance bin, the standard deviations were also calculated for each interchange type and plotted as half error bars.  Microsoft Excel, through its “solver” tool, was used also to estimate the goodness of fit of observed values to a hyperbolic equation [19]

## Results 

**Figure 1:**
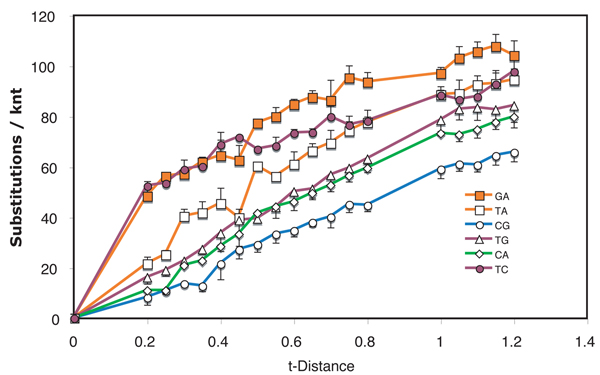
**Nucleotide substitution profile of tobamoviral genomes.** Levels of types of nucleotide interchanges between pairs of tobamoviral genome sequences at varying levels of divergence (t-distance).  Half error bars are standard deviations

**Figure 2:**
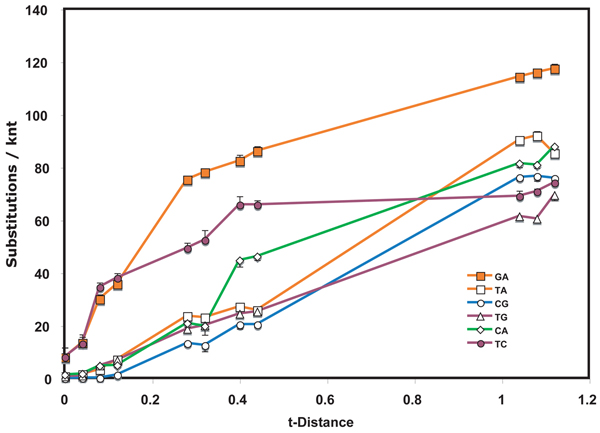
**Nucleotide substitution profile of *Wheat streak mosaic virus* genomes** See Figure 1 legend for details.

**Figure 3:**
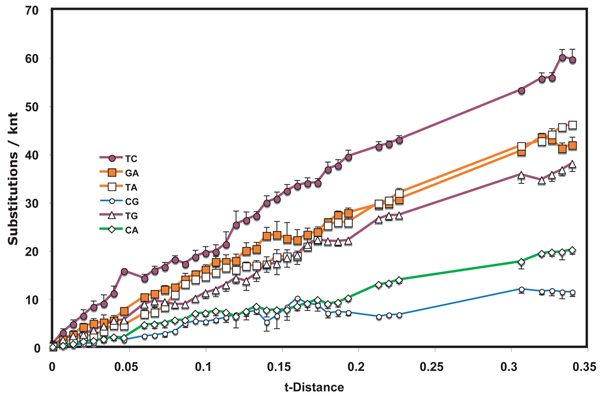
**Nucleotide substitution profile of *Tomato yellow leaf curl virus*** genomes See Figure 1 legend for details.

**Figure 4:**
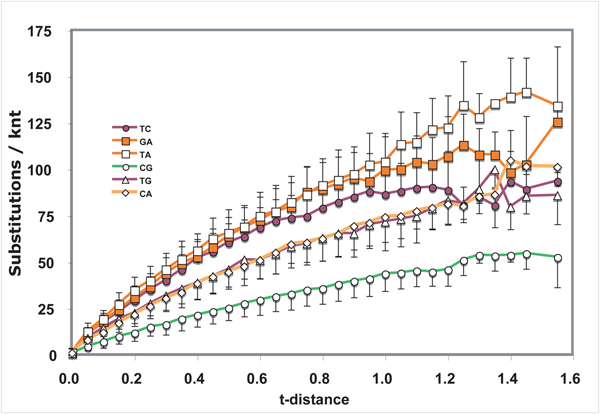
**Nucleotide substitution profile of retroelements of diverse eucaryotes** See Figure 1 legend for details.

**Figure 5:**
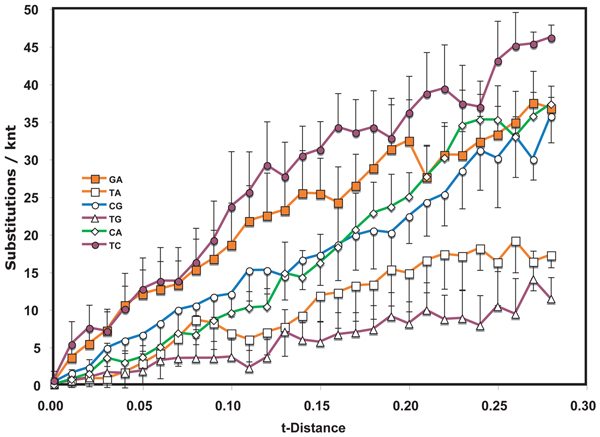
**Nucleotide substitution profile of RbcS coding regions from cereals** See Figure 1 legend for details.

**Figure 6:**
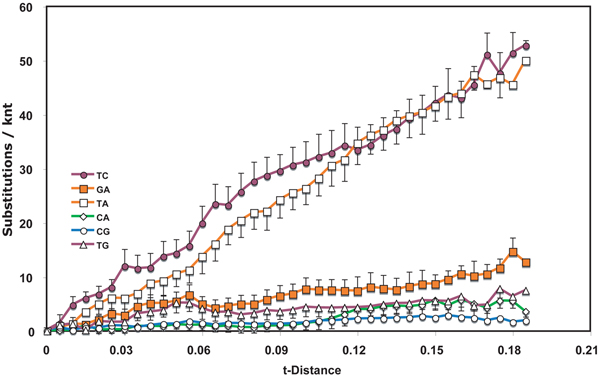
**Nucleotide substitution profile of RbcL coding regions from red algae** See Figure 1 legend for details.

Figs. 1, 2, 3, 4, 5, 6, show the accumulation of nucleotide interchanges between pairs of sequences as a function of the divergence t-distance between the sequences.  Absent other considerations, the relationships should be hyperbolic with an initial nearly linear phase followed by a leveling-off as previously interchanged sites undergo additional substitutions.  

### Viral interchange accumulation profiles

For the dataset of species from the *Tobamovirus* genus (Fig. 1), transitions accumulated 3.5 times as rapidly as transversions in the initial period of divergence.  On further accumulation of substitutions during divergence, the T<>C interchanges appeared to plateau at a lower level than G<>A interchanges. Transitions and transversions had similar rates of accumulation during this period of divergence.  At the largest divergence distances, the number of T<>A transversion interchanges could not be distinguished from the number of T<>C transition interchanges.  Not surprisingly, the complementary T<>G and C<>A interchanges had practically indistinguishable accumulation profiles, while C<>G interchanges were least frequent.  Between t-distances of 0.45 and 0.50, significant anomalous shifts in interchange frequency occurred for T<>A, G<>A and T<>C interchanges.

The *Tobamovirus* data set was chosen to focus on speciation events. For comparison, multiple isolates of the same virus species were tested for two viruses (Figs. 2 and 3). Limitation of the dataset to members of the same species resulted in a narrowing of the range of t-distances plotted.  For isolates of  WSMV (Fig. 2), transitions accumulated eight times as rapidly as transversions in the initial period of divergence.  Indeed, the transition accumulation curves appeared non-hyperbolic, being characterized by an apparent lag phase.  C<>G interchanges were rare during this period.  On further accumulation of substitutions, the T<>C interchanges appeared to plateau after more limited divergence times than did G<>A interchanges. Between t-distances of 0.12 and 0.28, a  significantly anomalous transient shift in interchange frequency occurred for C<>A interchanges.

WSMV is a virus with a single-stranded positive sense RNA genome.  To contrast with WSMV, TYLCV, a virus with a circular ambisense DNA molecule as genome, was chosen (Fig. 3).  Despite the virus’ DNA genome, mutation rates, both inferred [20] and experimentally estimated [21], have been reported to be of the order of those for RNA viruses. Lag phases were apparent for several inerchange types.  T<>C interchange accumulation was more prominent than others, including G<>A interchanges, in the initial period of divergence.  Indeed, accumulation of G<>A transition interchanges resembled closely that of T<>A transversions.  C<>G and C<>A transversion interchanges accumulated much less rapidly than transversions involving T.  Although C<>G interchanges appeared to have plateaued between 0.19 and 0.21 t-distance units, additional such interchanges were noted in more diverged pairs.

### Non-viral interchange accumulation profiles

The reverse transcriptase coding regions of nuclear retroelements from a variety of eukaryotes were assembled into a dataset to provide a highly diverged protein-coding gene set that may be under reduced selective pressure (Fig. 4).  With the exception of non-significant fluctuations at high divergence levels, the accumulation profiles of the different types of interchanges conformed to the expected hyperbolic pattern.  Correlation coefficients ranged from 0.988 for T<>C to 0.997 for C<>G interchanges.  T<>A transversion interchanges joined the two transitions (T<>C and G<>A) as the most frequent type of substitution in this dataset.  As with the viral profiles, C<>G interchanges accumulated to the smallest extent.

As additional controls, datasets of coding regions for plastid and nuclear subunits of ribulose-bisphosphate carboxylase (RbcL and RbcS, respectively) were also examined.  As for viral sequences, initial interchanges between recently diverged pairs of RbcS from cereals (Fig. 5) were 4.7 times as likely to be transitions than to be transversions and G<>A transitions began to plateau at a lower level of divergence distance than did T<>C transitions. T<>G interchanges were the least frequent at all levels of divergence and significantly less frequent than the complementary C<>A interchange, except at the very shortest divergence distances.

For RbcL coding regions from algae (Fig. 6), T<>C transition and T<>A transversion interchanges were the most prominent throughout the period of divergence, with T<>C exceeding T<>A except in the most diverged pairs.  At the highest divergence levels these two accounted for four-fold as many interchanges as for the other four combined.  As with the viral profiles, C<>G interchanges accumulated to the smallest extent.  An apparent anomalous change occurred between 0.11 and 0.12 t-distance units where C<>A transversion interchanges were more prominent among distant pairs than with less distant pairs.  This transition appeared to have been compensated by fewer T<>C interchanges at greater distances.

### Interchange comparisons

Table 1 compares the percentages of the total interchanges represented by each of the six types of interchange, obtained for the data sets analyzed in this study at a t-distance of 0.10, with two classic sets of data, one on globin genes [22]  and one on pseudogenes [23].  A t-distance of 0.1 was chosen for the comparison to avoid influence of highly frequent mutations (t-distance <0.5) and the influence of substitution saturation seen at higher t-distances. The classic percentages had been summed over all distances, but nonetheless provide a useful comparison.  For the pseudogenes, presumably subject to relaxed evolutionary constraints on substitutions, only G<>A transition interchanges were noticeably higher than other types.  G<>A interchanges were not the predominant type for five of the six datasets analyzed in the current study. The exception was the tobamoviral sequences where the G<>A percentage was slightly higher than that for T<>C. G<>C interchanges were relatively rare in all datasets except for globin and the RbcS ones. In globin genes their percentage was second only to the G<>A transition interchange.  The complementary substitution pair A<>C and G<>T had similar percentages in pseudogene, retroelement and *Tobamovirus* datasets, but were biased in the other substitution classes.

**Table 1:** Substitution Profiles of Selected Viral and Non-viral Genes

Substitution	Globin^a^	Pseudogenes^b^	rbcL^c^	Tobamov.^c^	rbcS^c^	Retroel.^c^	TYLCV^c^	WSMV^c^
G <> A	32.4	30.1	10.7	20.0	25.0	18.6	21.7	38.0
T <> C	13.1	9.2	42.9	18.2	31.8	19.7	26.4	40.4
A <> T	9.2	9.1	36.2	18.3	9.2	22.8	19.8	7.6
A <> C	14.5	11.5	2.0	15.1	12.8	14.4	9.6	5.3
G <> C	24.5	9.5	2.1	12.2	16.2	8.1	7.2	1.4
G <> T	6.5	10.5	6.1	16.2	5.0	16.4	15.2	7.4

^a ^[1]

^b ^[2]

^c ^At t-distance = 0.10; see Figs. 1, 2, 3, 4, 5, 6.

## Discussion

The interchanges summarized in this work are represented as X<>Y to make clear that no attempt has been made to determine the direction of substitutions, whether X changed to Y or Y changed to X.  Such determination requires confident knowledge of ancestral sequences.  This knowledge was lacking in most instances.  Trial runs using a consensus sequence as common ancestor resulted in separation of the six curves into twelve curves.  Often the reciprocal exchange values were widely different.  Investigation into mechanisms responsible for anomalies and differences will need to analyze the full set of 12 types.  However, such separation was not needed to observe the existence of anomalies.

If substitutions were to occur with equal frequency on coding and non-coding strands, one would expect that, because of complementarity, T<>A and C<>G interchanges would be detected equally frequently as would C<>A and T<>G interchanges.  T<>A  exchanges were observed to be prominent in the tobamovirus, retroelement and TYLCV datasets, while C<>G interchanges were minor in all but the RbcS dataset.  On the other hand, C<>A and T<>G interchange levels had practically indistinguishable accumulation profiles in the tobamovirus dataset.  This was also true for retroelements, RbcL and, at low to moderate divergence levels, for WSMV, but not for TYLCV or RbcS. 

In the viral datasets, among sequence pairs that had undergone only limited divergence, one or both transition interchanges were by far the most predominant type.  For tobamoviruses and WSMV both transitions had this feature while for the TYLCV only T<>C interchanges showed this property.  The same transitions played a much diminished role in pairs that diverged from one another over a longer period of time.  Similar early spurts of T<>C interchanges occurred also in evolution of both Rbc coding regions, with G<>A interchanges also being prominent early in RbcS.  The result suggests that there are subsets of sites that can readily tolerate transitions and that substitutions at these sites approach saturation after only short periods of evolution.  If the suggestion is correct, then such changes in substitution frequency need to be taken into account when calibrating phylogenetic trees to determine dates of divergence.  Initial rates of divergence may be substantially higher than those after saturation of these sites has been reached.

Of greatest relevance to the issue of dating viral phylogenetic trees is the nature of the curves representing the accumulation of substitutions over evolutionary time.  Fig. 4 shows that, for a dataset where selective pressures are likely to have been slight during evolution, the curves are very consistent with the expected hyperbolic relationships.  The other datasets generated graphs with irregular appearances, including lag phases and abrupt changes in slope.  It is intriguing that these slope changes appear to occur at specific divergence levels.  That they may represent substitutions associated with speciation events merits investigation.  They are likely associated with a shift in base composition. The apparent lag phases are likely the result of a non-linear relationship between t-distance and time.  Between recently diverged pairs some types of interchanges (usually transitions) happen frequently at a limited number of sites.  These interchanges occur over a small time span thus artificially expanding the x-axis at early “times” of divergence and resulting in apparent lag phases.  This interpretation is consistent with the view that substitution frequencies are much higher for intrastrain divergence than for interspecies divergence.  Thus, it may not be valid to apply substitution frequencies calculated from recently diverged pairs to evolution of species.

## Conclusions

These observations suggest strongly that the probabilities of particular substitutions are different at different stages of evolution.  Thus, a single probability applied to an entire dataset, such as those analyzed for Figs. 1, 2, 3 and 5, 6 should produce unreliable results in phylogenetic analysis. This study therefore supports the conclusion [10] that different evolutionary rules may apply to recent divergences than apply to divergences that give rise to speciation and similar distant past events.

## Competing interests

The author declares that he has no competing interests.

## Supplementary Material

Additional file 1 Accession and gi numbers of sequences used in the study Click here for file

Additional file 2 File, when unzipped, consists of a folder with aligned fasta files for each of the six datasets employed in this study. Click here for file

Additional file 3 Code, when compiled in Future Basic, which was used to calculate numbers of interchanges of each type for pairs of aligned sequences.   Output was input to Microsoft Excel for further processing. Click here for file

## References

[B1] Duffy S Shackelton LA Holmes EC Rates of evolutionary change in viruses: patterns and determinants. Nat Rev Genet 2008 9 267 276 10.1038/nrg2323 18319742

[B2] Harkins G Delport W Duffy S Wood N Monjane A Owor B Donaldson L Saumtally S Triton G Briddon R Experimental evidence indicating that mastreviruses probably did not co-diverge with their hosts. Virol J 2009 6 104 10.1186/1743-422X-6-104 19607673 PMC2719613

[B3] Gibbs A Evolution and origins of tobamoviruses. Philos Trans R Soc Lond B Biol Sci 1999 354 593 602 10.1098/rstb.1999.0411 10212939 PMC1692536

[B4] Gibbs A Po T Liang-yi K Ying-chun T Randles J Classification of several tobamoviruses isolated in China on the basis of the amino acid composition of their virion proteins. Intervirology 1982 18 160 163 10.1159/000149319 7141834

[B5] Lartey RT Voss TC Melcher U Tobamovirus evolution: gene overlaps, recombination, and taxonomic implications. Mol Biol Evol 1996 13 1327 1338 8952077 10.1093/oxfordjournals.molbev.a025579

[B6] Kang HJ Bennett SN Sumibcay L Arai S Hope AG Mocz G Song JW Cook JA Yanagihara R Evolutionary insights from a genetically divergent hantavirus harbored by the European common mole (Talpa europaea). PLoS One 2009 4 e6149 10.1371/journal.pone.0006149 19582155 PMC2702001

[B7] Wu B Melcher U Guo X Wang X Fan L Zhou G Assessment of codivergence of mastreviruses with their plant hosts. BMC Evol Biol 2008 8 335 10.1186/1471-2148-8-335 19094195 PMC2630985

[B8] Koonin EV Wolf YI Nagasaki K Dolja VV The complexity of the virus world. Nat Rev Microbiol 2009 7 250 19234475

[B9] Ramsden C Holmes EC Charleston MA Hantavirus evolution in relation to its rodent and insectivore hosts: no evidence for co-divergence. Mol Biol Evol 2008 26 143 153 10.1093/molbev/msn234 18922760

[B10] Gibbs AJ Fargette D Garcia-Arenal F Gibbs MJ Time--the emerging dimension of plant virus studies. J Gen Virol 2010 91 13 22 10.1099/vir.0.015925-0 19889925

[B11] Swofford DL Olsen GJ Waddell PJ Hillis DM Hillis DM Moritz C, Mable BK Phylogenetic inference. Molecular Systematics 1996 2 Sunderland, Masssachusetts: Sinauer Associates 407 514

[B12] Gojobori T Ishii K Nei M Estimation of average number of nucleotide substitutions when the rate of substitution varies with nucleotide. J Mol Evol 1982 18 414 423 10.1007/BF01840889 7175958

[B13] Thorne JL Kishino H Felsenstein J An evolutionary model of maximum likelihood alignment of DNA sequences. J Mol Evol 1991 33 114 124 10.1007/BF02193625 1920447

[B14] Drummond AJ Rambaut A BEAST: Bayesian evolutionary analysis by sampling trees. BMC Evol Biol 2007 7 214 10.1186/1471-2148-7-214 17996036 PMC2247476

[B15] Pond SLK Frost SDW Muse SV HyPhy: hypothesis testing using phylogenies. Bioinformatics 2005 21 676 679 10.1093/bioinformatics/bti079 15509596

[B16] Xia X Xie Z Salemi M, Vandamme A-M Tetrapod phylogeny and data exploration using DAMBE. The Phylogenetic Handbook 2003 Cambridge: Cambridge University Press

[B17] Se-Al http://tree.bio.ed.ac.uk/software/seal/

[B18] Jukes TH Cantor CR Munro MN Evolution of protein molecules. Mammalian Protein Metabolism 1969 III New York: Academic Press

[B19] John EG Simplified curve fitting using spreadsheet add-ins*. Int J Engng Ed 1998 14 375 380

[B20] Duffy S Holmes EC Phylogenetic evidence for rapid rates of molecular evolution in the single-stranded DNA begomovirus tomato yellow leaf curl virus. J Virol 2008 82 957 965 10.1128/JVI.01929-07 17977971 PMC2224568

[B21] Ge L Zhang J Zhou X Li H Genetic structure and population variability of tomato yellow leaf curl china virus. J Virol 2007 81 5902 5907 10.1128/JVI.02431-06 17376922 PMC1900275

[B22] Gojobori T Li WH Graur D Patterns of nucleotide substitution in pseudogenes and functional genes. J Mol Evol 1982 18 360 369 10.1007/BF01733904 7120431

[B23] Li WH Wu CI Luo CC Nonrandomness of point mutation as reflected in nucleotide substitutions in pseudogenes and its evolutionary implications. J Mol Evol 1984 21 58 71 10.1007/BF02100628 6442359

